# Investigation of Single-Wall MoS_2_ Monolayer Flakes Grown by Chemical Vapor Deposition

**DOI:** 10.1007/s40820-015-0064-2

**Published:** 2015-10-05

**Authors:** Nihan Kosku Perkgoz, Mehmet Bay

**Affiliations:** Department of Electrical and Electronics Engineering, Faculty of Engineering, Anadolu University, 26555 Eskisehir, TR Turkey

**Keywords:** Monolayer, Chemical vapor deposition, Two-dimensional materials, Molybdenum disulfide (MoS_2_)

## Abstract

Recently, two-dimensional monolayer molybdenum disulfide (MoS_2_), a transition metal dichalcogenide, has received considerable attention due to its direct bandgap, which does not exist in its bulk form, enabling applications in optoelectronics and also thanks to its enhanced catalytic activity which allows it to be used for energy harvesting. However, growth of controllable and high-quality monolayers is still a matter of research and the parameters determining growth mechanism are not completely clear. In this work, chemical vapor deposition is utilized to grow monolayer MoS_2_ flakes while deposition duration and temperature effect have been systematically varied to develop a better understanding of the MoS_2_ film formation and the influence of these parameters on the quality of the monolayer flakes. Different from previous studies, SEM results show that single-layer MoS_2_ flakes do not necessarily grow flat on the surface, but rather they can stay erect and inclined at different angles on the surface, indicating possible gas-phase reactions allowing for monolayer film formation. We have also revealed that process duration influences the amount of MoO_3_/MoO_2_ within the film network. The homogeneity and the number of layers depend on the change in the desorption–adsorption of radicals together with sulfurization rates, and, inasmuch, a careful optimization of parameters is crucial. Therefore, distinct from the general trend of MoS_2_ monolayer formation, our films are rough and heterogeneous with monolayer MoS_2_ nanowalls. Despite this roughness and the heterogeneity, we observe a strong photoluminescence located around 675 nm.

## Introduction

As the dimensions of materials are reduced from three dimensions (3D), the fundamental physical properties change remarkably, allowing for novel applications, which are otherwise not possible [1, 2]. Transition from 3D to two dimensions (2D) first became possible with the exfoliation of graphene, a material well-known for its high electron mobility, mechanical strength, and thermal conductivity [3–6]. Unfortunately, there are still important challenges involved in the transition of graphene from laboratories to industry especially due to its zero bandgap [7]. Therefore, researchers are still in search of a novel material system preferably with a direct bandgap to be used in electronic, photonic, and energy applications. In this regard, transition metal dichalcogenides (TMDC), MX_2_ (*M* = Mo, W; *X* = S, Se, Te) are receiving special attention with their peculiar optical and electronic properties [8–10] and their potential to be used in catalysis, microelectronics, batteries, hydrogen storage, medical applications, and optoelectronics [11–14]. Among the numerous candidates, monolayer MoS_2_ has invoked a particular interest due to its direct bandgap and it is considered to have great potential both in electronics and photonics [15–17]. MoS_2_-based field effect transistors (FETs) used as phototransistors, memory devices, and sensors have shown superior properties including excellent mobility, ON/OFF ratio, and sensitivity [18]. Also, MoS_2_ has attracted attention due to its thermal properties and its potential use in thermoelectric applications [18, 19] and catalyzing properties resulting from its active edge sites [20]. However, scalable, controllable, large-area growth of single-layer MoS_2_, keeping high crystal quality and large domain size, still remains a problem [14, 21]. Mechanical exfoliation, one of the most commonly used methods, is not favorable for commercial applications. Likewise, liquid exfoliation, ionic intercalation, and hydro-thermal methods still have important drawbacks where batch fabrication and device applications are concerned [22–25]. It is also possible to form different few-layer TMDCs through colloidal synthesis techniques, which are beneficial in terms of high-yield and substrate-free nanostructures [26]. However, developing a facile and reliable method for large-area growth is crucial in order to use such 2D materials in the different applications of electronics and photonics.

One method which seems particularly promising is chemical vapor deposition (CVD), as it is highly promising in its ability to grow monolayer, controlled, large-area MoS_2_ films [14, 21, 27–29]. CVD-based synthesis was first reported in 2012 [21, 22], showing the potential of the method to realize high-quality, controlled growth of the MoS_2_ flakes, and other researches have revealed that monolayer flakes deposited by CVD can be used in different applications and devices such as phototransistors [30], photodetectors [31], memories [16], and so on. Although the underlying mechanism of the monolayer MoS_2_ growth is still not very clear, it can allow for determining the features of the thin films through controlled synthesis [32–34].

In the present work, we have used CVD to obtain single-wall monolayer MoS_2_ flakes whose features can be changed through process parameters. We report the direct influence of deposition duration and process temperature on the growth mechanism and film properties. By changing process parameters, we can shift from a rough surface with MoS_2_ flakes scattered at different angles to a smoother and more uniform surface composed of monolayer MoS_2_ formations. Such single-wall MoS_2_ flakes, which for the most part are not flat on the surface, can be beneficial in applications including solar cells [35], energy storage [36], catalysis [11], and sensing [37] where large surface area of the flakes can increase the intended performance.

## Experimental

**Fig. 1:**
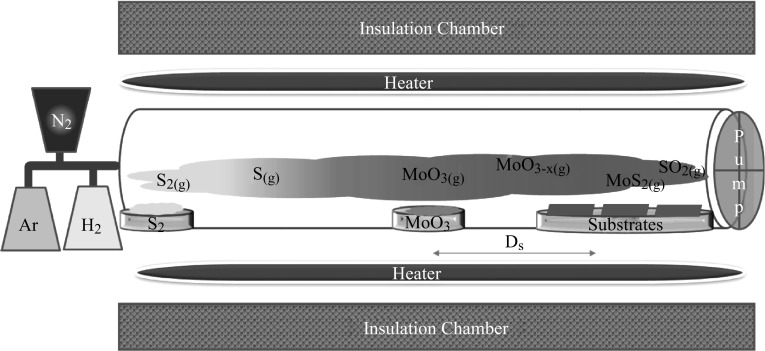
The chemical vapor deposition system for the deposition of MoS_2_ flakes

The experimental setup of the CVD system is schematically shown in Fig. 1. The quartz boats containing high-purity MoO_3_ (14 mg, 99.9 %, Aldrich) and S powder (1.4 g, 99.5 %, Alfa) were placed at the temperature zones specified according to the melting temperatures of the precursors, 700 °C for MoO_3_ and 150 °C for sulfur. The MoO_3_ boat was located at the highest temperature zone of the furnace. In our setup, spatial locations have been critical for complete sulfurization and monolayer MoS_2_ formation as the temperature changes with distance. This effect was investigated by varying the distance between boats of MoO_3_ powder and the substrates (*D*_s_) from 9 cm (~670 °C) to 13 cm (~600 °C). The SiO_2_ (~300 nm)-coated Si substrates are cleaned using piranha etch solution and the RCA technique. SiO_2_ was thermally grown on Si, and the thickness is confirmed by ellipsometry. Before deposition started, the reaction chamber was heated to deposition temperature at a rate of 15 °C min^−1^ in a nitrogen environment, and at the specified deposition temperatures, Ar and H_2_ were introduced to the system as carrying gases at flow rates of 17 and 10 sccm, respectively. The growth duration was decreased from 10 to 5 and 3 min to observe the evolution of the flakes.

At deposition temperatures, S vapor reduced MoO_3_ powder to volatile suboxide MoO_3−x_ [38] and MoS_2_ monolayer flakes were formed by the gas-phase reactions while the compounds diffusing on the substrate reacting with sulfur are obtained possibly through the Eqs. (1) and (2) [21]. It is suggested that if the reaction duration is not sufficient, the sulfurization process cannot be fully accomplished and intermediate products are formed, one of which is MoO_2_, due to its stability [38]. After the growth period was finished, a rapid cooling-down process was carried out and 500 sccm of nitrogen gas (99.999 %) was purged into the tube to avoid other products such as MoO_3_ formation while keeping MoS_2_ monolayers on the surface of the substrate.

1 $${\text{MoO}}_{ 3} + {\text{(x/2)S}} \to {\text{MoO}}_{{ 3-{\text{x}}}} + {\text{(x/2)SO}}_{ 2},$$

2 $${\text{MoO}}_{{ 3-{\text{x}}}} + {\text{(7}-\text{X)/2}}\;{\text{S}} \to {\text{MoS}}_{ 2} + {\text{(3} - \text{X)/2}}\;{\text{SO}}_{ 2}.$$

The MoS_2_ monolayer formations were characterized by Raman spectroscopy, photoluminescence (PL), scanning electron microscopy (SEM), and high-resolution transmission electron microscopy (HRTEM). In the case of MoS_2_ deposition, Raman spectroscopy is a practical and facile way to examine the film properties including the number of layers and the imprints of other possible products such as MoO_3_, MoO_3-x_, and MoO_2_ [39–42]. In bulk MoS_2_, there are two distinctive Raman peaks corresponding to in-plane vibration of Mo and S atoms ($E_{{2{\text{g}}}}^{1}$) at ∼383 cm^−1^ and the out-of-plane vibration of S atoms (*A*_1g_) at ∼408 cm^−1^ where the number of layers are determined by the change in difference between these two peaks [41]. As the number of layers decrease, the mode at $E_{{2{\text{g}}}}^{1}$ is found to move to lower frequencies and the mode at *A*_1g_ is found to move to higher frequencies [22, 39], giving valuable information about monolayer and multi-layer MoS_2_ flakes. Additionally, these peaks also shift with the change in film properties, the frequency of $E_{{2{\text{g}}}}^{1}$ vibrational mode is found to be effected by strain [43], and the frequency of *A*_1g_ vibrational mode is found to depend on electrostatic doping [44]. PL also gives valuable information about the transition from bulk or multi-layer indirect-bandgap MoS_2_ to direct-bandgap, few-layer or monolayer MoS_2_ flakes. For the PL measurements, a focused excitation laser (532 nm) was used to detect the peaks at ~670 nm (*A*_1_ excitation of MoS_2_) and ~630 nm (the resonance of B_1_ excitation) for MoS_2_ films [45]. At about 645 nm, a peak which is the sign of bilayer MoS_2_ can also be observed [46]. While the peak at ~630 nm is related with rather thick flakes as the layers go from multi-layer to monolayer MoS_2_, the peak around 670 nm gets more intense and sharper [47]. In addition to number of layers, photoluminescence also depends on various parameters including grain sizes, defects, strain, and electrostatic doping [27, 32].

SEM was utilized to investigate the shapes of the flakes and see the general view of the film formations. HRTEM was used a supportive technique for further understanding about the shapes of the flakes. In addition to this analysis, investigation of defect states and vacancies is important as they affect the transport mechanism and PL of the system [48–51]. However, in this research work, we rather focused on the properties of the nanowall film formations and their dependence on the process parameters.

## Results and Discussion

**Fig. 2:**
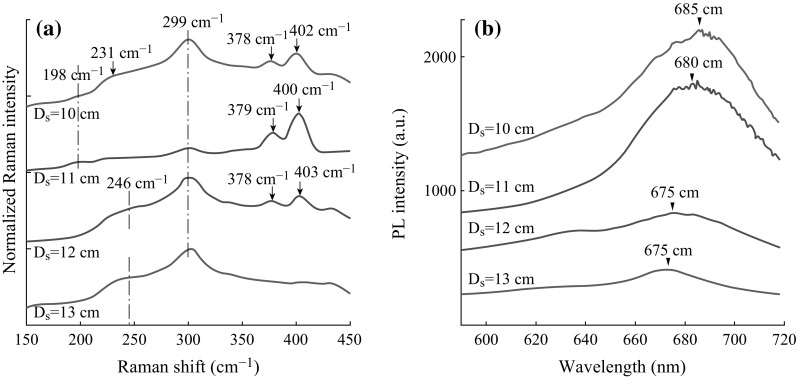
**a** Raman scattering and **b** PL spectra of the films grown on SiO_2_ coated Si substrate for *D*_s_ values between 10 and 13 cm while the reaction period is fixed to 10 min

MoS_2_ flakes have been grown using solid MoO_3_ and S as precursors at different spatial locations, abbreviated as *D*_s_ (distance between MoO_3_ powder boat and the substrates) as illustrated in Fig. 1. As shown in Fig. 2a, b, Raman scattering (excitation wavelength at 532 nm) and PL spectra are primarily analyzed to identify the film characteristics including the number of MoS_2_ layers and formation of other products different from MoS_2_ [52, 53]. In the first set of experiments, *D*_s_ is changed from 10 to 13 cm while the deposition time is fixed to 10 min. The change in the distance can be indicative of the change in precursor concentration [34]. However, with our setup (Fig. 1), this effect can be ignored where the substrate temperature (*T*_S_) changing with the distance is suggested to be a rather more effective parameter to influence the film properties.

As presented in Fig. 2a, although the two distinctive MoS_2_ Raman peaks (around ∼379 and ∼402 cm^−1^ corresponding to in-plane vibration of Mo and S atoms according to the Raman peaks) are present, we also observe Raman signatures from oxide phases (MoO_3_ and/or MoO_x_) which indicate the presence of residual oxygen associated with incomplete sulfurization. The peak at ~299 cm^−1^ is an indicator of nanometer-thick MoO_3_ sheets (as small as ~15 nm), and it can be correlated with the stoichiometry of MoO_3_ polycrystalline samples or a shift of an initially strong 284 cm^−1^ peak [54, 55]. At *D*_S_ = 10 cm, a small peak located at 231 cm^−1^ is observed, most probably due to one-dimensional MoO_2_ nanorods [45], and at *D*_S_ = 11 cm, another weak peak centered around 198 cm^−1^ is explained by the formation of thick MoO_3_ sheets (~300 nm) [54]. As the distance (*D*_s_) becomes larger than 12 cm, the peak at 246 cm^−1^ starts to be more visible, indicative of $B_{\text{g}}^{3}$ twist mode, showing that the MoO_3_ nanoparticles get larger at lower substrate temperatures [54]. We can suggest that when the temperature is not sufficiently high, the activation energy of the reactions in Eq. (2) is not provided. In fact, from the Raman peaks, measured from the films formed at a distance of 10 cm (*T*_S_ of ~655 °C), the imprints of MoO_3_ nanoparticles are also observed. In this case, it is quite probable that the diffusion of the sulfur species is degraded and formation of MoO_3_ nanoparticles is more likely than the formation of MoS_2_ flakes. These results show that balancing desorption–adsorption and sulfurization rates is crucial to obtain high-quality, monolayer MoS_2_ flakes. Obviously, the network that we obtained is not homogeneous, and there are different MoO_x_ (*X* = 2, 3) sheets and rods being formed during the deposition, which also helps the MoS_2_ flakes to be suspended on the surface allowing for a larger surface area.

MoO_3_ and/or MoO_x_ peaks are ignorable only for the sample deposited at *D*_s_ = 11 cm (*T*_S_ of ~655 °C), which suggests that the deposited MoS_2_ flakes are rather uniform and high quality with an optimized substrate temperature of precursor ratio of MoO_3_ to S. This can be explained by the efficient adsorption and diffusion of MoO_3-x_ species on the substrate and reaction with S to form MoS_2_ at around 640 °C. Although other substrate temperature regimes also have been reported for MoS_2_ formation [14, 33], it is concluded that due to the complicated nucleation process, it is possible to find different optimal conditions to grow monolayer MoS_2_ flakes in different systems. Therefore, depending on the system geometry, flow rates, and other process parameters, the substrate temperature should be carefully controlled to fine-tune the adsorption/desorption of radicals and the sulfurization rate for the growth of MoS_2_ monolayers [56]. Our results show that the difference in Raman peaks is between 21 and 25 cm^−1^ corresponding to in-plane vibration of Mo and S atoms ($E_{{2{\text{g}}}}^{1}$) at ∼378–379 cm^−1^ and the out-of-plane vibration of S atoms (*A*_1g_) at ∼400–403 cm^−1^. Both $E_{{2{\text{g}}}}^{1}$ and *A*_1g_ modes exhibit a blueshift when compared to bulk MoS_2_ in agreement with other nanowall-like MoS_2_ layers [57]. However, the peak convergence is less than that of the reported monolayer MoS_2_ results in the literature, which can be explained by the fact that the network is not completely composed of monolayer MoS_2_ formations, but the film, specifically the parts closer to the substrate surface, contains oxysulfides (MoOS_2_), MoO_x_ (*X* = 2, 3), and multi-layer flakes [51, 58, 59]. High-temperature conditions (*D*_s_ = 10 cm) are considered unfavorable for MoS_2_ monolayer formation due to their high diffusion rate, which obstructs the growth of stable nuclei.

As shown in Fig. 2b, although their full width at half maximum (FWHM) is not narrow, the PL peaks between ~675 and ~685 nm indicate formation of monolayer MoS_2_ flakes, specifically at a distance (*D*_s_) of 11 cm. At this specified spatial location, more homogeneous and rather large-size MoS_2_ mono layers are formed and a stronger photoluminescence develops, which results from the direct excitonic transition due to the direct bandgap of monolayer MoS_2_ [47]. For the films deposited at other substrate temperatures, the intensity of peaks becomes lower and their FWHM becomes wider. In general, the formed structures are not uniform, and, especially at low temperatures, the roughness and heterogeneity increase.

**Fig. 3:**
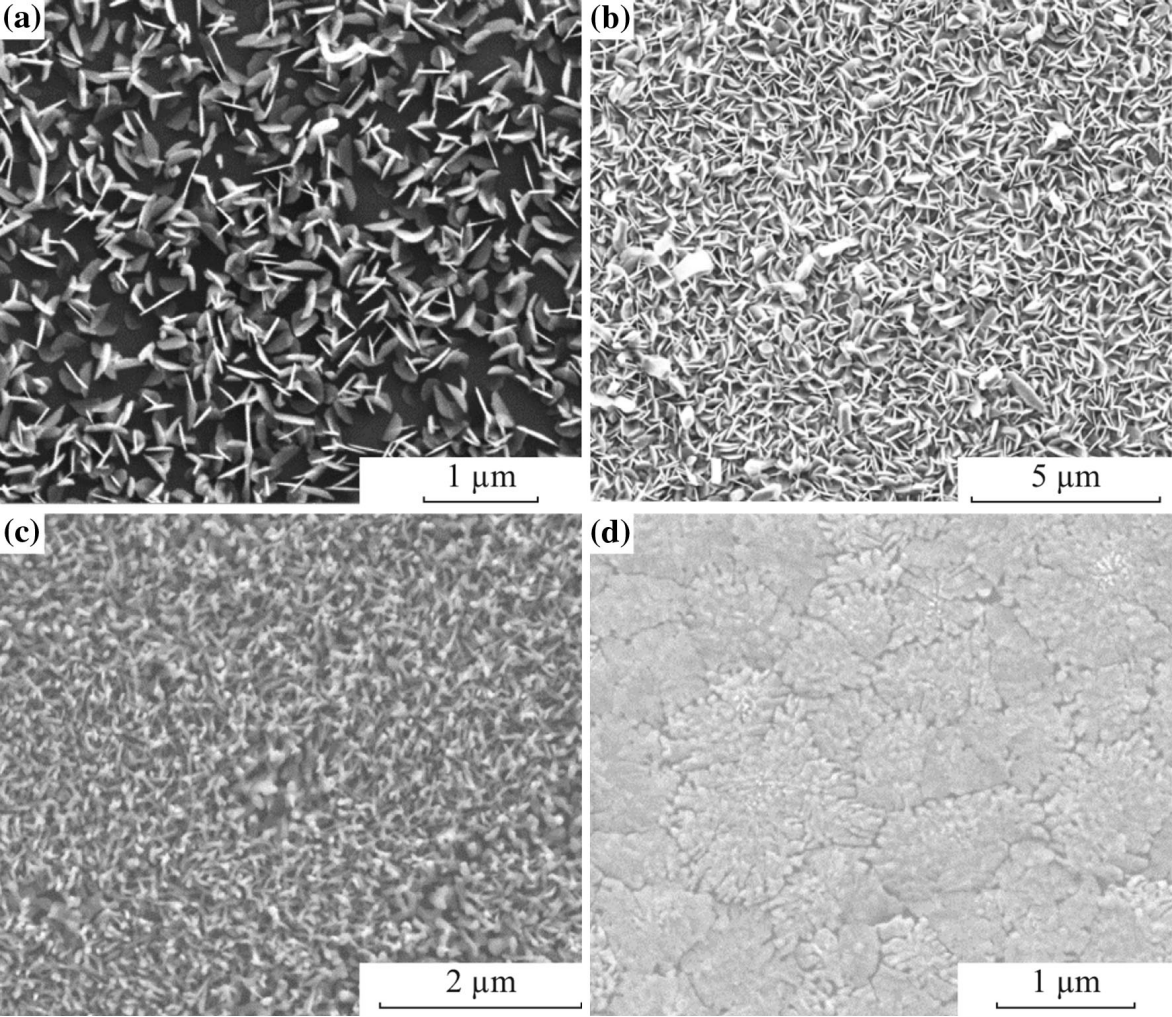
SEM Images for the films deposited when *D*_s_ = **a** 10 cm, **b** 11 cm, **c** 12 cm, and **d** 13 cm with a reaction period of 10 min

Figure 3 exhibits the SEM pictures of the films in Fig. 2, giving information about the size, shape of the flakes, and general network of the formations. These images show features similar to nanowalls and nano-plates that are edge oriented due the basal edges deposited on the substrate [60]. Such nanowall-like structures are appealing for their potential to be used as super capacitors, specifically in Li-ion batteries [36, 61–63]. Also, high surface area can be beneficial when used in heterostructures where non-radiative energy transfer is utilized. Rather high crystal growth rate and large number of nucleation sites are considered to provide smaller crystalline grain sizes. As a result, the formed surface is not homogeneous and the surface is far from being smooth. When the substrate temperature is reduced (at *D*_s_ = 13 cm), the surface roughness decreases and such nanowall-like structures become less visible (Fig. 3d).

**Fig. 4:**
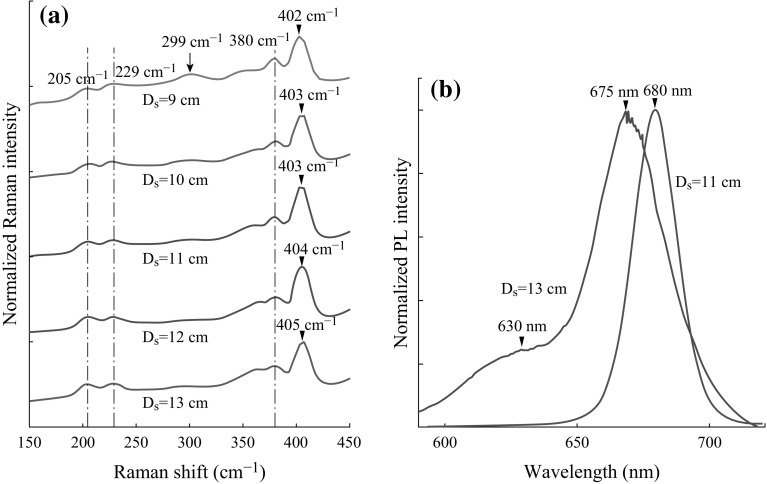
**a** Raman scattering spectra for films deposited at *D*_S_ values between 9 and 13 cm; **b** PL spectra for the films deposited at *D*_S_ = 11 and 13 cm while the reaction period is fixed to 5 min

It is expected that process duration and the concentration of the reactants also play a crucial role in the formation of MoS_2_ films. Therefore, to get a better understanding of the growth mechanism, we decreased the deposition duration from 10 to 5 min. Figure 4a, b shows Raman scattering and PL spectra, respectively. Indeed, both the Raman scattering and PL spectra exhibit that a more homogeneous network is obtained along with a much reduced amount of other types of formations such as MoOS_2_, MoO_2_, or MoO_3_. The peak at ~299 cm^−1^, an indicator of nanometer-thick MoO_3_ sheets. has nearly disappeared, and the difference in MoS_2_ Raman peak has become as small as ~22 cm^−1^. This Raman peak difference, higher than expected, could be explained with a large number of small-size grains or crystalline defects. Different from the Raman scattering spectra measured from the films deposited in 10 min, another peak at ~355 cm^−1^ starts to develop, indicative of m-MoO_2_ b-MoO_3-x_ phase transition [64]. This suggests that in 5 min, S vapor has reduced MoO_3_ powder to volatile suboxide MoO_3-x_ [32] and in 10 min, these MoO_3-x_ regions have already been transformed into either MoS_2_ monolayer flakes or MoO_3_ nanoparticles (see Fig. 2a). As the substrate distances get larger, a peak around 365 cm^−1^, assigned to O–Mo–O bending and scissoring modes, also becomes more visible [65].

It should be noted that there is also a significant improvement in the PL characteristics of the films deposited in 5 min (Fig. 4b) compared to the films with a reaction period of 10 min (Fig. 2b). This improvement is observed specifically for the films that are deposited at a *D*_S_ of 11 cm (T_S_ of ~640 °C). MoS_2_ flakes are mostly in the form of single layers, which is understood from the disappearing peak at ~ 630 nm, an indicator of multi-layer flakes. Also, the peak at ~675 shifts to ~680 nm and becomes narrower, showing higher-quality and homogeneous flakes at a *D*_s_ of 11 cm compared to the case at a *D*_s_ of 13 cm. The PL spectra at 9, 10, and 12 cm are not included in the Figure because they do not show a significant difference from the peak obtained from the film deposited at the *D*_S_ of 13 cm. In general, these results explain that sulfurization in the first 5 min is sufficient; however, as the deposition period is increased, other products start to develop. It is obvious that optimizing the precursor concentrations is also crucial to prevent molybdenum di/three oxide sheets and allows for the formation of large MoS_2_ monolayers, and these parameters are specific to the experimental setup and deposition temperature. As described by Wang et al., edge free energy and the ratio (or amount) of the precursors in the medium are the determining factors for the growing rate and hence the shape of the grains [34].

**Fig. 5:**
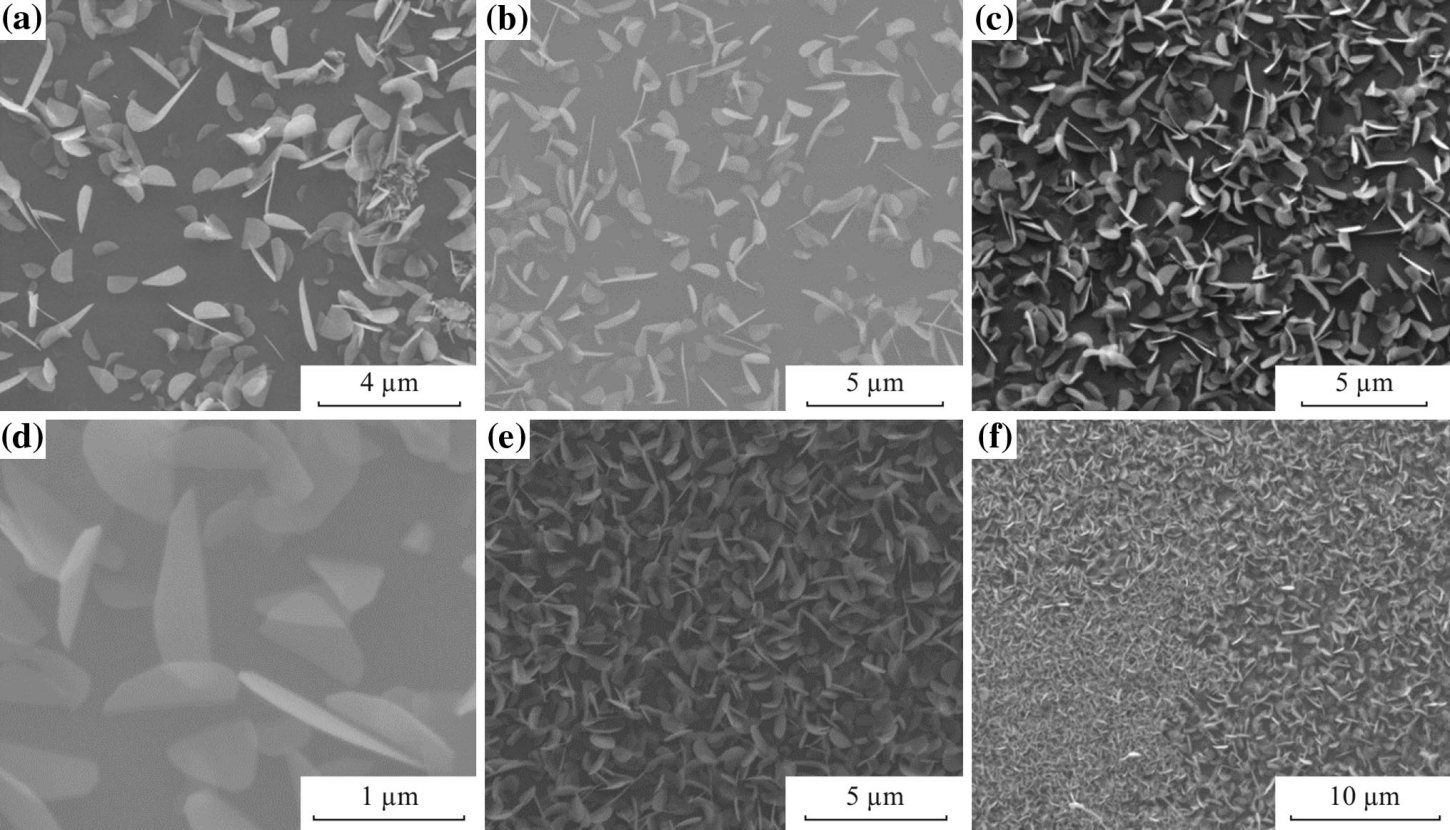
SEM Images for CVD MoS_2_ on SiO_2_-coated Si substrate when *D*_s_ = **a** 9 cm, **b** 10 cm, **c**, **d** 11 cm, **e** 12 cm, and **f** 13 cm with a reaction period of 5 min

Figure 5 presents the SEM images of these films formed in 5 min. Different from the films formed in 10 min, the flakes are visible specifically when deposited at a *D*_s_ of 9, 10, and 11 cm. Commonly, it is discussed that the domain shape of the flakes tend to grow in the form of a triangle or a hexagon [34]. However, most of our MoS_2_ flakes are in the shape of half pringles resembling truncated triangles where the shapes of domains are suggested to be controlled by the ratio of precursors effecting the kinetic growth dynamics of edges [27, 58]. SEM images also show that flakes are not flat on the surface of the substrate but rather they appear to stand erect with different angles. These observations confirm that gas-phase reactions are effective in our system making MoS_2_ flakes round-shaped and erect on the surface instead of being flat on the surface.

**Fig. 6:**
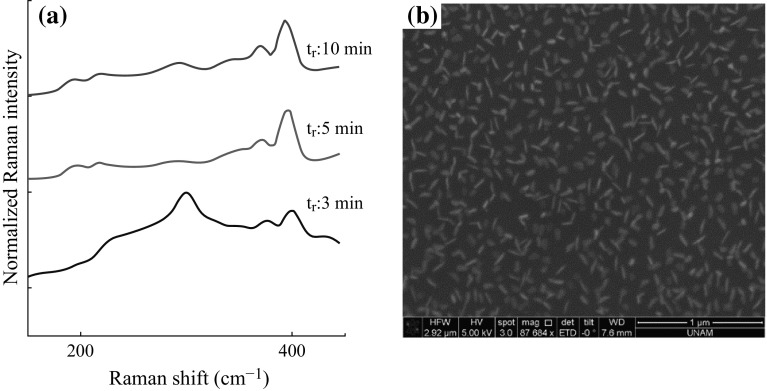
**a** Raman spectra for CVD MoS_2_ on SiO_2_-coated Si substrate when the reaction period is fixed to 3, 5, and 10 min when *D*_S_ is fixed to 11 cm; **b** SEM image with a reaction period of 3 min

Observing the improvement in the formation of MoS_2_ monolayers with a decreased deposition duration of 5 min with respect to the case of 10 min, this period is further reduced to 3 min. Figure 6a shows Raman scattering spectra of the films formed in 3, 5, and 10 min when *D*_s_ is fixed to 11 cm. Interestingly, the peak at ~299 cm^−1^ has again become visible, showing that nanometer-thick MoO_3_ sheets are formed on the surface. This result is due to insufficient deposition duration for MoS_2_ flakes to form. Figure 6b confirms that the flakes are relatively small when compared with the ones in Fig. 5.

**Fig. 7:**
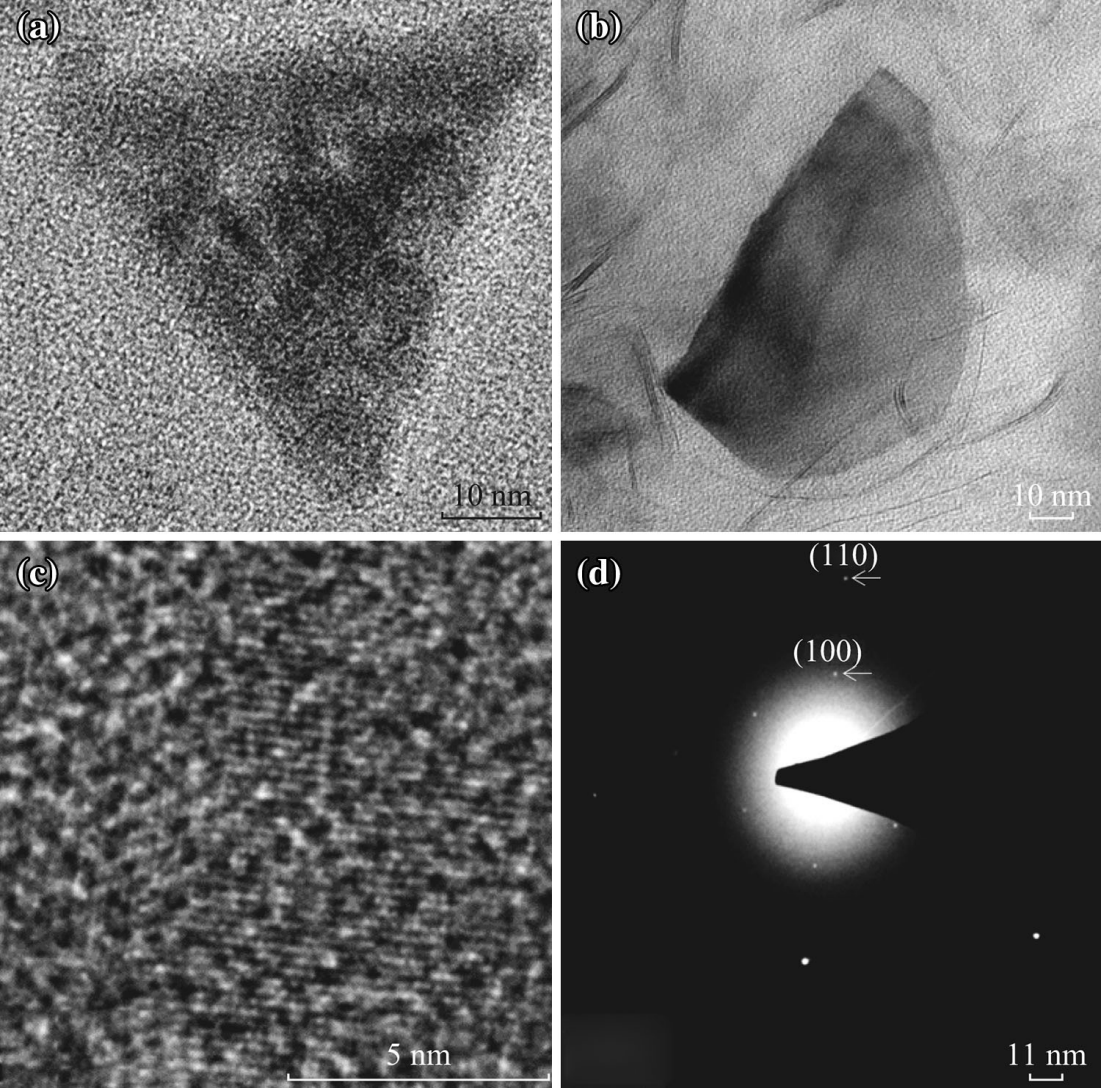
TEM Image of **a** triangular and **b** chips-like shapes **c** periodic atomic arrangement, **d** diffraction patterns of a monolayer triangular flake

We investigated selected-area electron diffraction and HRTEM (FEI TECNAI G2 F30 model) images with an accelerating voltage of 200 kV to have further understanding of flake structures. The HRTEM image in Fig. 7a, b exhibits a monolayer MoS_2_ triangular and “chips-like” shape where Fig. 7c shows the periodic atomic arrangement. As it can be observed from the SEM images (Figs. 3, 5), most of the flakes are “chip-like” in shape, and triangular-shaped flakes also exist in the film network. Figure 7d displays diffraction patterns of a monolayer triangular flake. These single flakes have been prepared using the as-grown films through simply rinsing in water and drop casting the solution on a TEM grid. The symmetry in the diffraction spots show that the flake is a continuous single crystal, and there are no rotational boundaries. Corresponding selective area electron diffraction (SAED) pattern with [001] zone axis shows hexagonally arranged diffraction spots assigned to the (100) and (110) planes [21] suggestive of the highly crystalline nature of the MoS_2_ flakes.

## Conclusions

We have shown that when substrate temperature and growth duration are controlled, MoS_2_ flakes with different properties including nanowall-like rough surfaces and relatively smoother monolayer MoS_2_ surfaces can be grown. Also, side products such as MoO_3_ and MoO_2_ can be avoided by fine-tuning the process parameters. Due to the complicated nucleation and growth process, it is important to optimize these parameters in different system geometries and flow rates to adjust the adsorption/desorption of radicals obtain sufficient sulfurization rates and find optimal conditions to grow monolayer MoS_2_ flakes. In our experiments, in addition to triangular flakes, we obtained rounded flakes resembling truncated triangles, which are not flat on the surface but rather erect at different angles. This result suggests that gas-phase reactions are also effective in monolayer MoS_2_ formation. Despite the roughness and heterogeneity of the formed network, we obtained films exhibiting a strong PL peak, a characteristic behavior of monolayer flakes, due the erect monolayer MoS_2_ nanowalls. These results show that such single-wall MoS_2_ flakes, which are mostly not flat on the surface, possess a rather larger surface area and can be beneficial in applications such as solar cells, energy storage, catalysis, and sensors with higher performance.
